# Supplementary figures and images for: Tracking conformational transitions of the gonadotropin hormone receptors in a bilayer of (SDPC) poly-unsaturated lipids from all-atom molecular dynamics simulations
Source: PLoS Comput Biol. 2024 Jan 11;20(1):e1011415. doi: 10.1371/journal.pcbi.1011415 (PMC10807830; doi:10.1371/journal.pcbi.1011415)

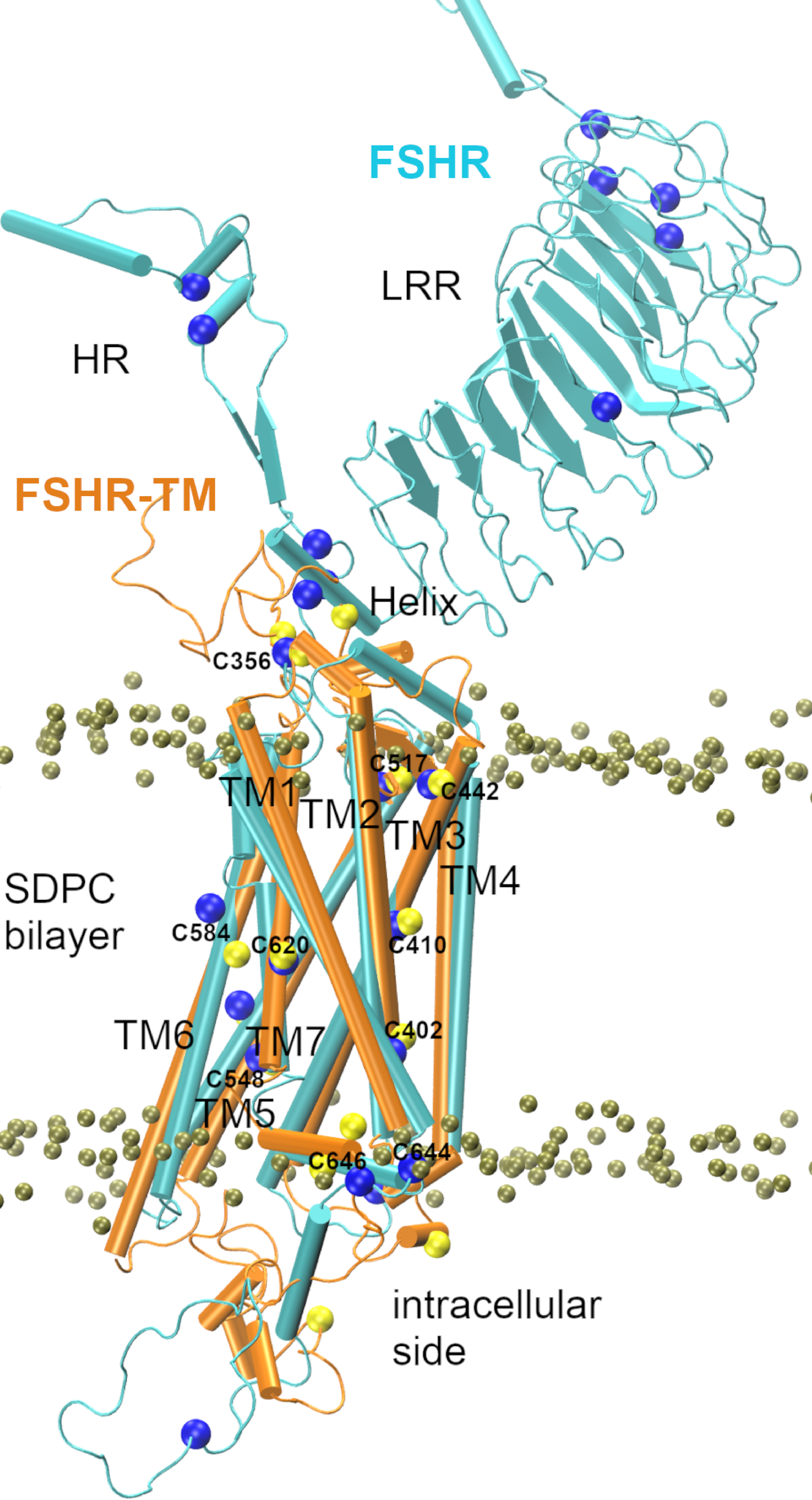

Supplement: S1 Fig — (TIF) [file pcbi.1011415.s001.tif]

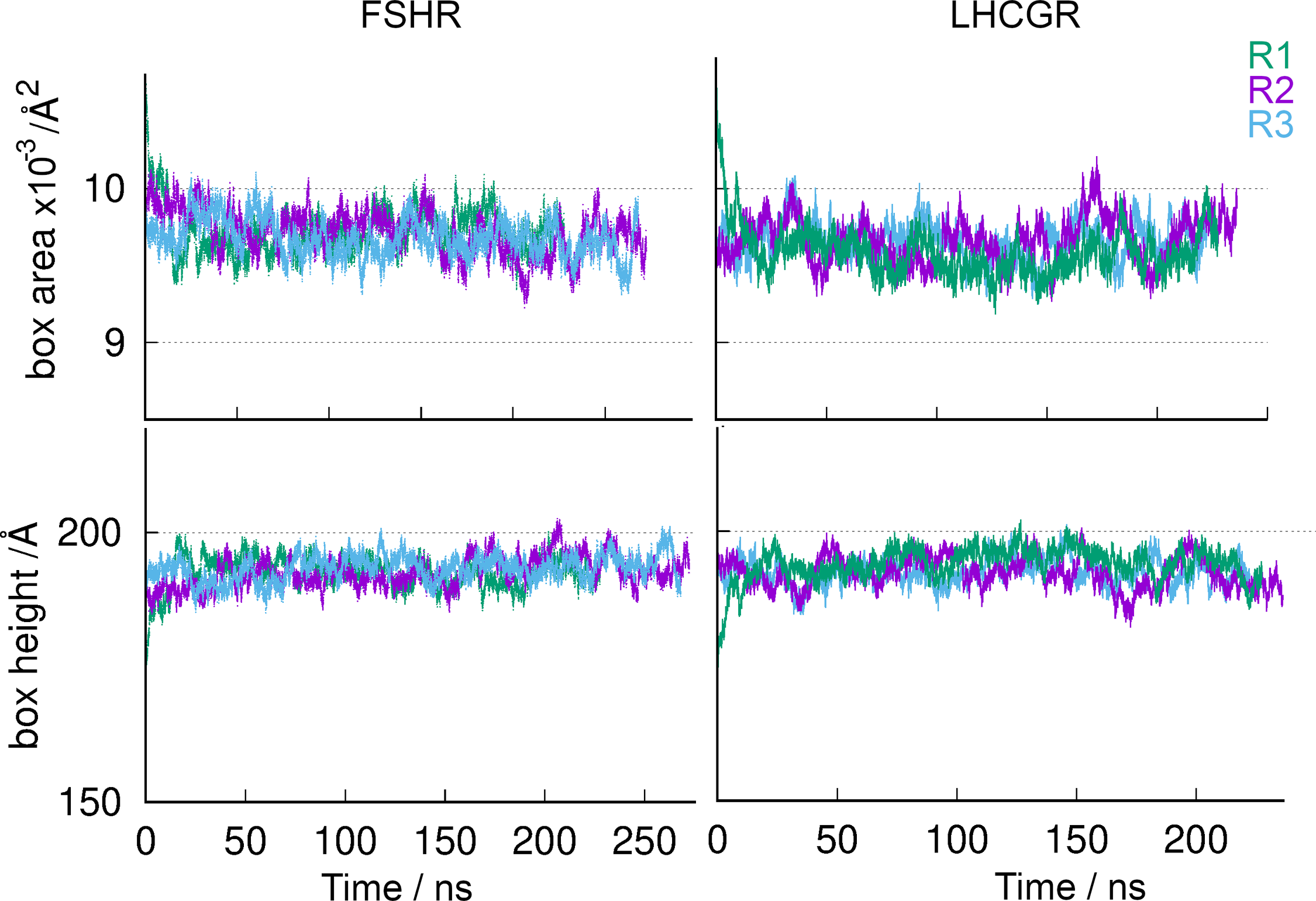

Supplement: S2 Fig — Stable averages were calculated after 50 nanoseconds for box area (xy-dimensions) and box height (z-dimension), both parameters important for monitoring the equilibration of the membrane bilayer according to the area per lipid and hydrophobic height in SDPC lipid molecules. (TIF) [file pcbi.1011415.s002.tif]

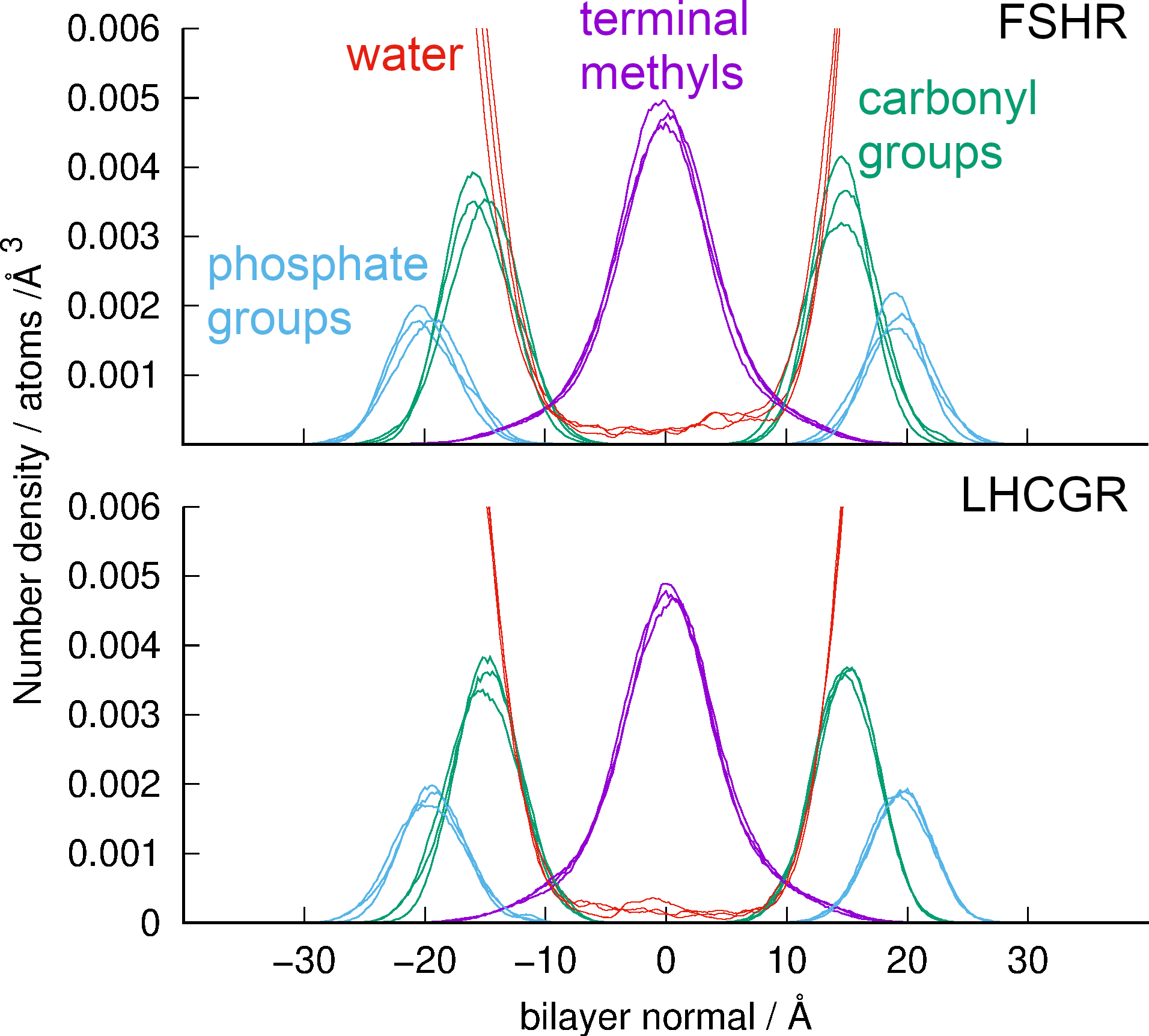

Supplement: S3 Fig — Water and SDPC functional groups were identified in both, FSHR (top panel) and LHCGR (bottom panel) systems. Calculation were performed for the last 40 ns of trajectory for replicates R1-R3. Distributions are normalized to the number of atoms/Å3. (TIF) [file pcbi.1011415.s003.tif]

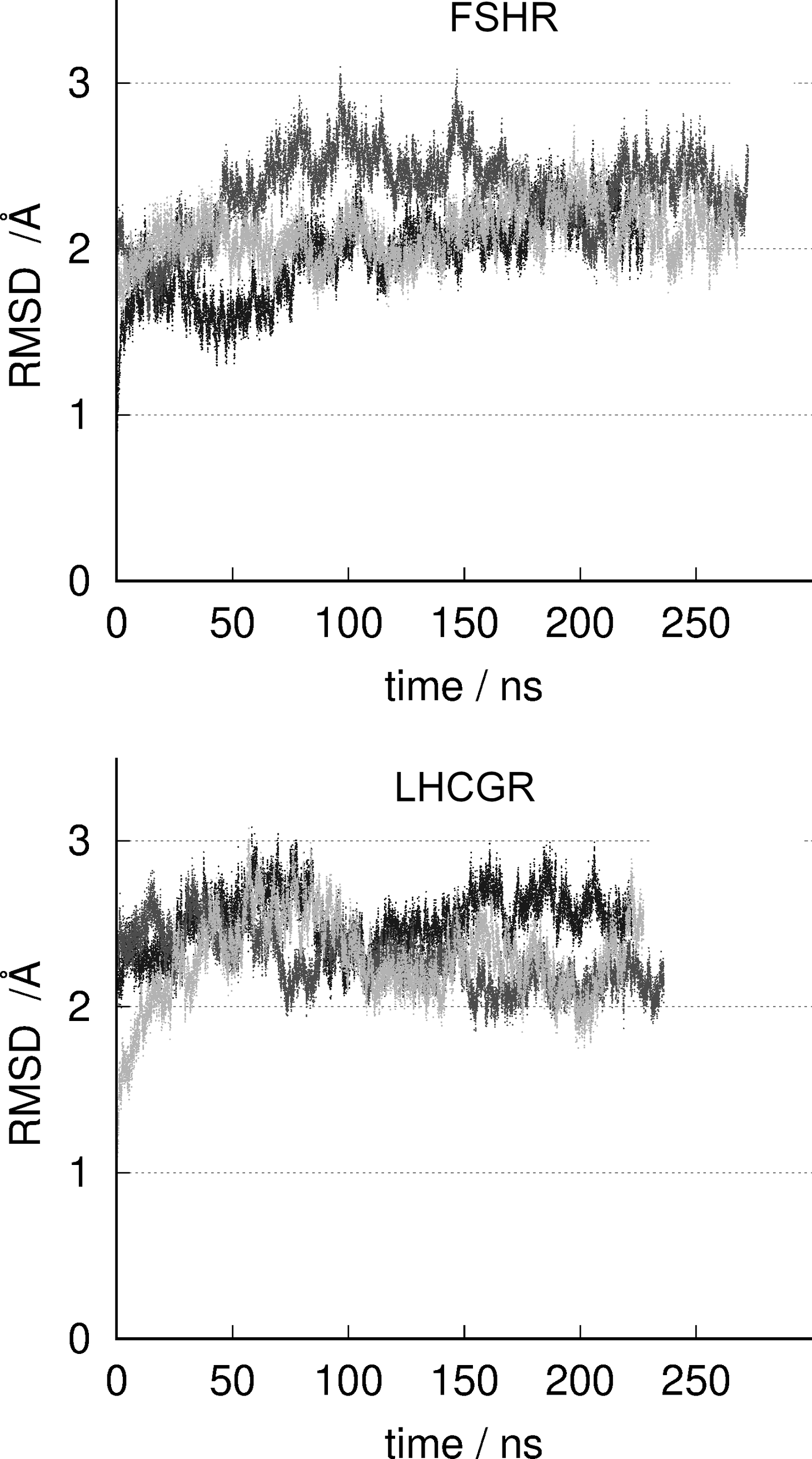

Supplement: S4 Fig — Root mean squared displacement (RMSD) calculations for the Cα atoms of the transmembrane domains in FSHR (top panel) and LHCGR (bottom panel). Results for R1-R3 replicates were included. (TIF) [file pcbi.1011415.s004.tif]

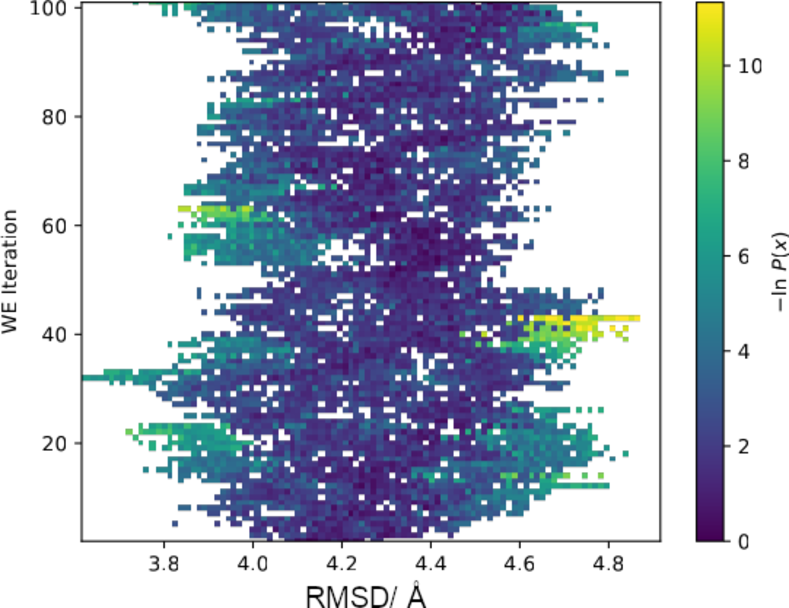

Supplement: S5 Fig — The inactive state was the reference for the RMSD calculation (TM domain). Variability of the RMSD over 100 iterations of 200 ps trajectories populated bins in the interval 3.6 to 4.9 Å. Up to 14 simultaneous trajectories per bin were generated in the simulation to sample states with low probability. (TIF) [file pcbi.1011415.s005.tif]

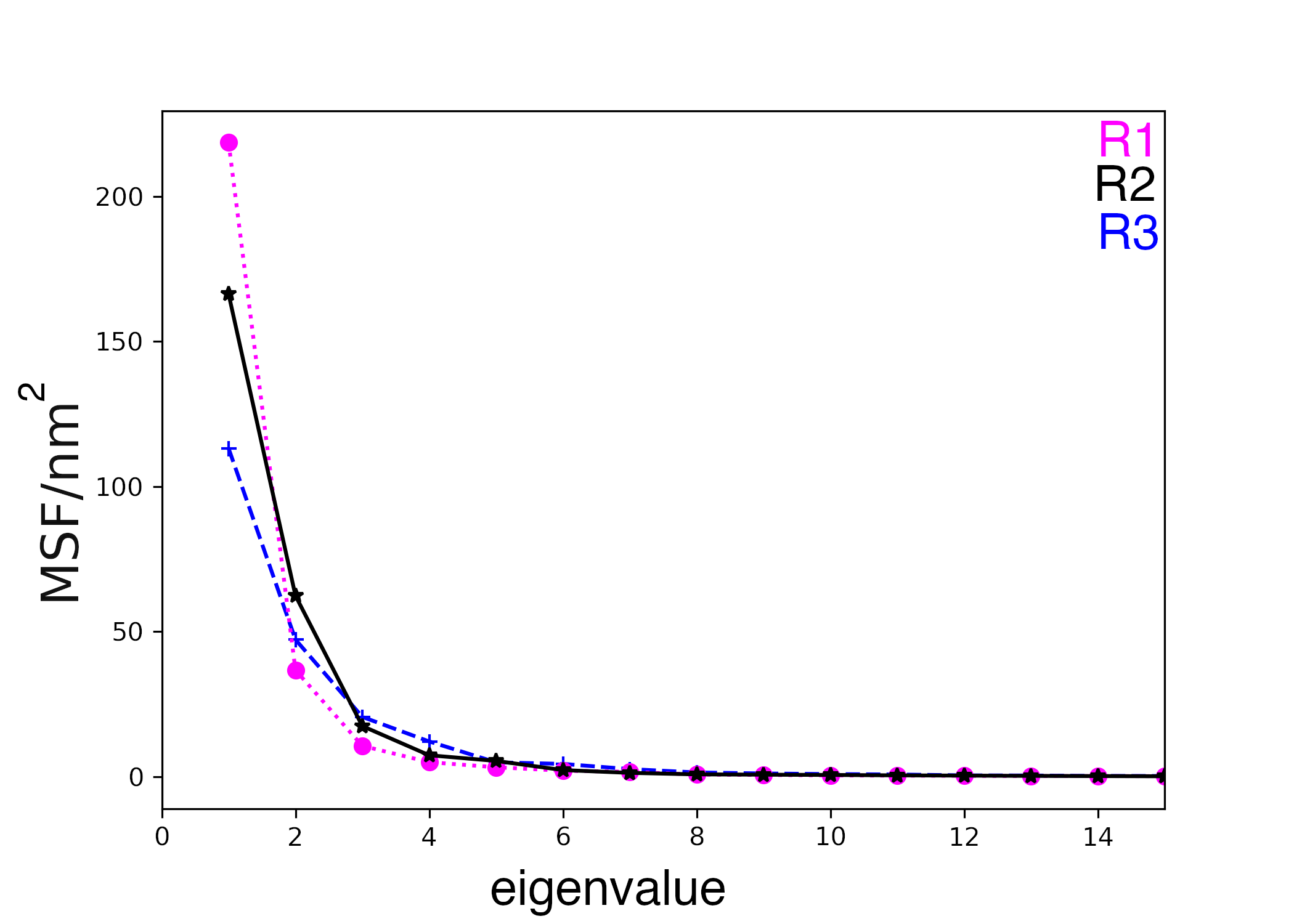

Supplement: S6 Fig — The total fluctuation corresponds to the sum of eigenvalues. Contribution of eigenvalues 1 to 4 accounts for the ~95% of root mean squared fluctuation. (TIF) [file pcbi.1011415.s006.tif]

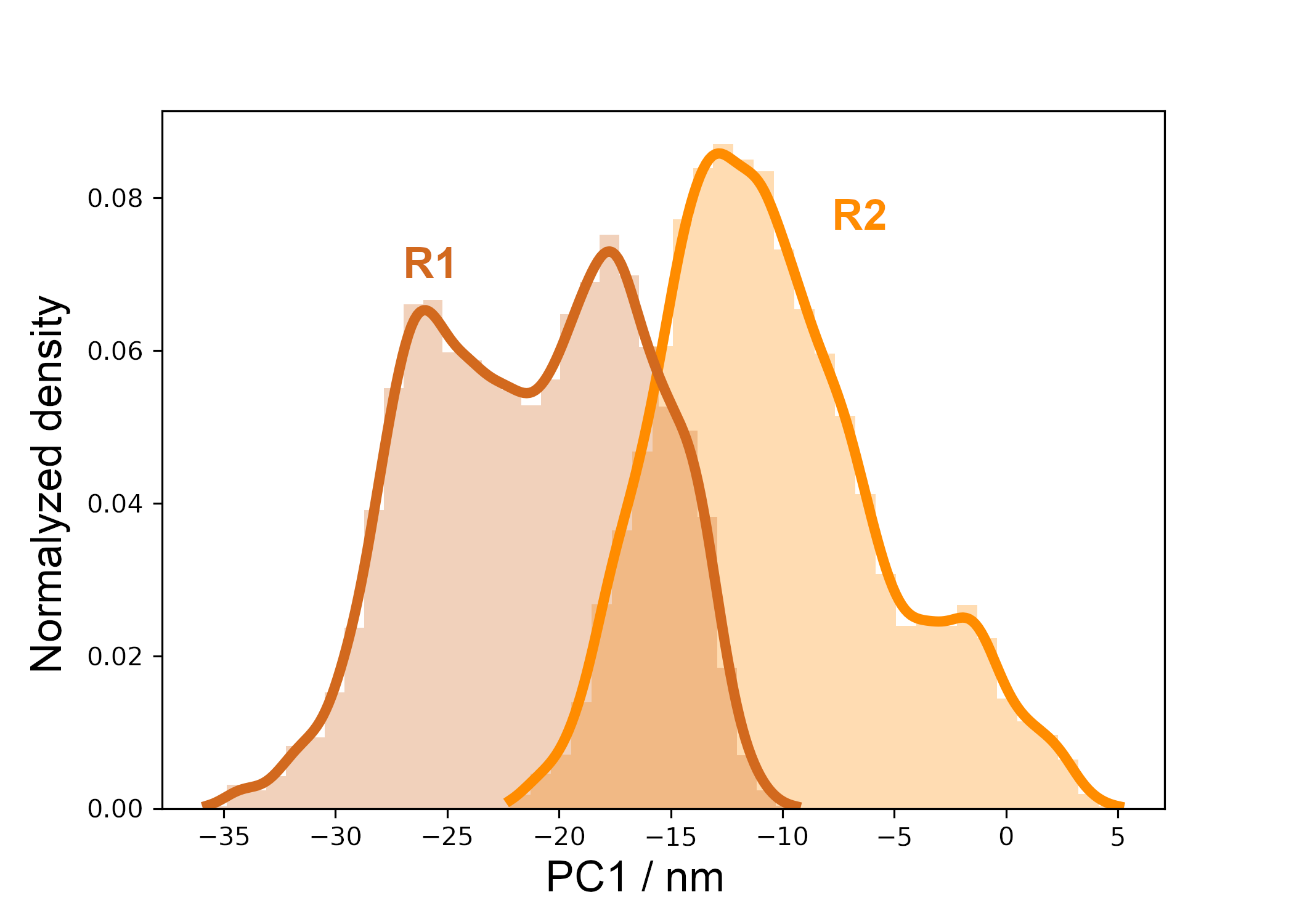

Supplement: S7 Fig — For comparison, the trajectory of R1 was projected over the first eigenvector of R2. The overlap of distributions represents conformations with similar motion (fluctuations) in both trajectories, otherwise conformations display different dynamics due to complementary sampling in independent replicates. (TIF) [file pcbi.1011415.s007.tif]

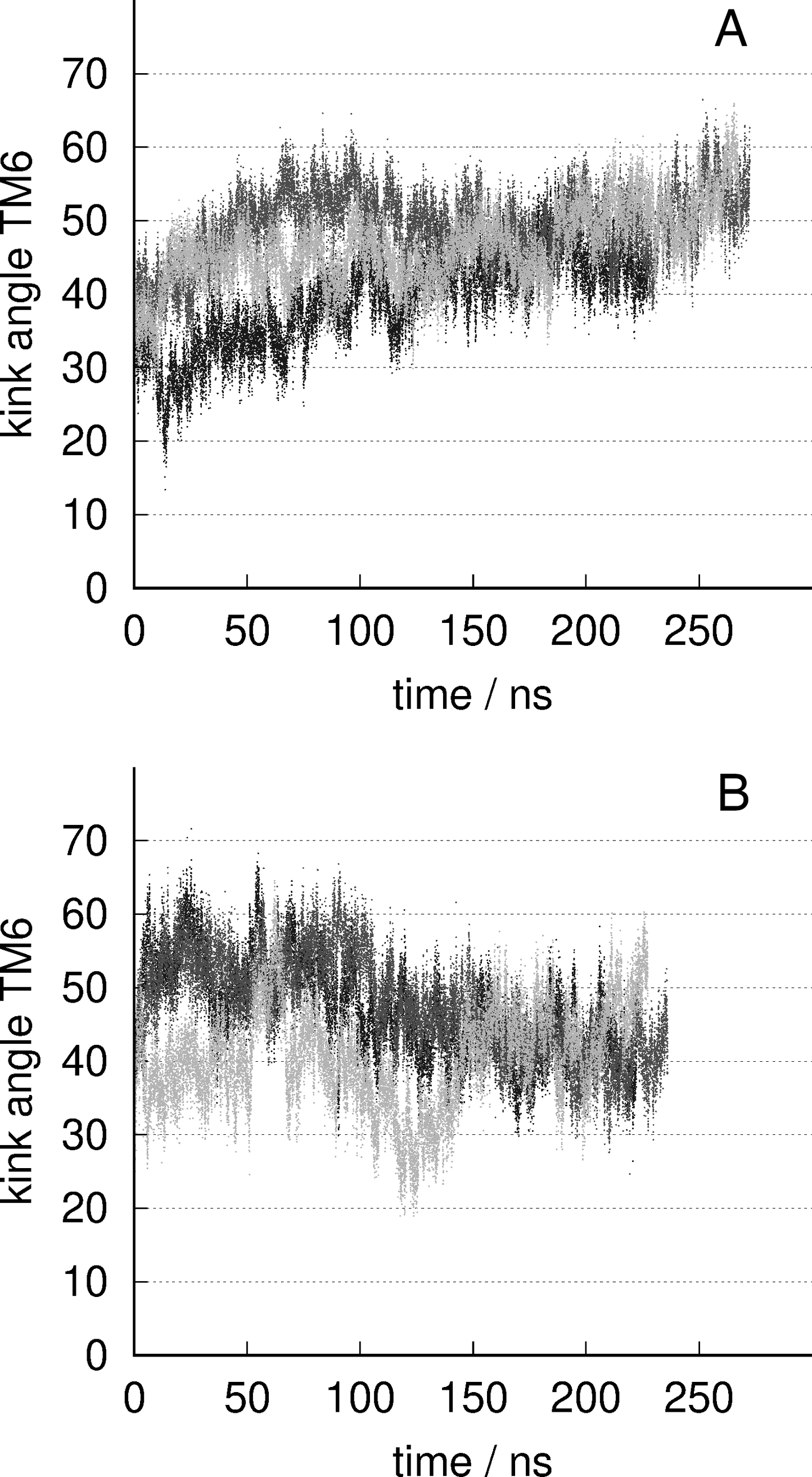

Supplement: S8 Fig — A. Calculation for FSHR using positions of Ca atoms of L597 and M858 for the upper half, D581 and S565 for the lower half, and a reference value of 44 deg. B. Calculation for LHCGR using Ca atoms of F594 and S586 for the upper half, D578 and C563 for the lower half, and reference value of 37 deg. (TIF) [file pcbi.1011415.s008.tif]

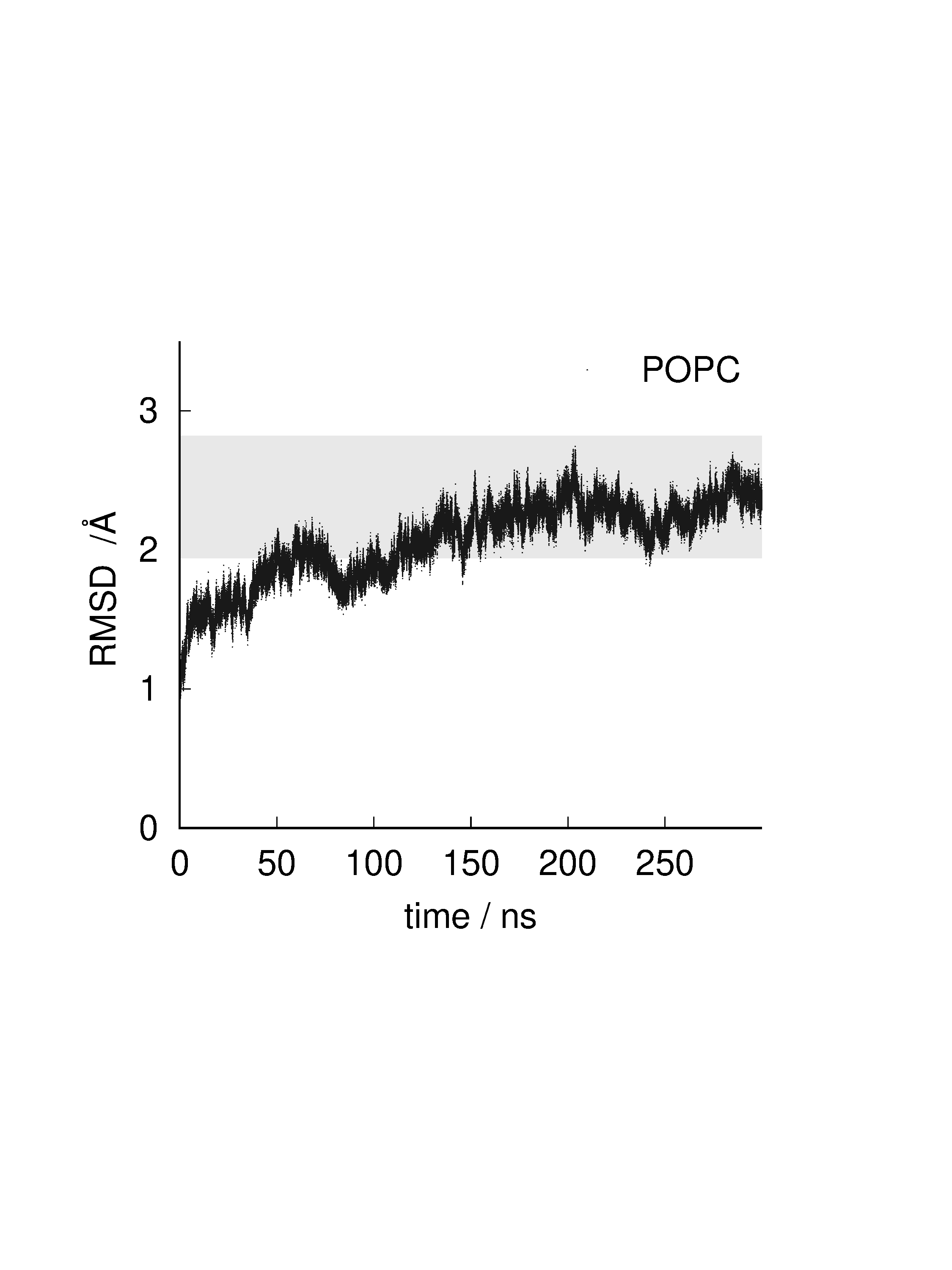

Supplement: S9 Fig — (TIFF) [file pcbi.1011415.s009.tiff]
