# Supplementary material for: Tracking conformational transitions of the gonadotropin hormone receptors in a bilayer of (SDPC) poly-unsaturated lipids from all-atom molecular dynamics simulations
Source: PLoS Comput Biol. 2024 Jan 11;20(1):e1011415. doi: 10.1371/journal.pcbi.1011415 (PMC10807830; doi:10.1371/journal.pcbi.1011415)
Supplement: S1 Table — Calculation for the last 164 ns of the R1-R3 trajectories in FSHR and LHCGR. Standard deviations calculated employing the blocking average method. (DOCX) [file pcbi.1011415.s013.docx]

**S1 Table**. Average of kink angles at TM helix 6. Calculation for the last 164 ns of the R1-R3 trajectories in FSHR and LHCGR. Standard deviations calculated employing the blocking average method [1]

| Run | FSHR | LHCGR |
| --- | --- | --- |
| R1 | -2.02±1.12 | 3.31±2.12 |
| R2 | 5.64±0.77 | 7.82±1.48 |
| R3 | 4.03±1.49 | 8.71±2.02 |

[1] Flyvbjerg H, Petersen HG. Error estimates on averages of correlated data. JChemPhys. 1989;91:461-6. PubMed PMID: 20532
