# Supplementary material for: Tracking conformational transitions of the gonadotropin hormone receptors in a bilayer of (SDPC) poly-unsaturated lipids from all-atom molecular dynamics simulations
Source: PLoS Comput Biol. 2024 Jan 11;20(1):e1011415. doi: 10.1371/journal.pcbi.1011415 (PMC10807830; doi:10.1371/journal.pcbi.1011415)
Supplement: Attachment — Submitted filename: Response_to_Reviewers.docx [file pcbi.1011415.s014.docx]

Reviewer #1: In this manuscript the authors present the results of a set of relatively short (200ns) uncorrelated MD simulations of large complex and highly flexible glycoprotein hormone receptors with the aim of understanding functional conformational transitions that can inform towards allosteric control mechanisms upon ligand binding.

I find the subject interesting, but unfortunately no evidence is provided to support that either system, which were started from a low 4.8 Å resolution cryo-EM (or no resolution, in case of the AF) conformations, can be considered at or near conformational equilibrium. The computational sampling used in the work is limited in its sampling potentials and for that reason not likely to bring such large and complex systems near conformational equilibrium. This is important as the system should be conformationally stable before data production and collection, for the results to be statistically representative and the insight functionally relevant.

As evidence of system´s stability we calculated the structural parameters for both, the membrane bilayer and the transmembrane domains of the receptors. In Fig. 1 we show the box dimensions, xy-area and z-height for the trajectories generated in the NPT ensemble. The box dimensions are relevant because fluctuations of the box size were set anisotropic for a proper equilibration of the membrane, since the membrane structure relies significantly on the area per lipid and hydrophobic core height. Box dimensions reached stable averages within 50 ns of trajectory.

Fig 1. Stability of the membrane bilayer in replicates R1-R3 for the FSHR and LHCGR systems. Stable averages were calculated after 50 nanoseconds for box area (xy-dimensions) and box height (z-dimension), both parameters are important for monitoring the equilibration of the membrane bilayer according to the area per lipid and hydrophobic height in SDPC lipid molecules.

In addition, the membrane equilibration was determined by the of mass distributions whereby molecular domains can be identified. The membrane topology encompasses the hydrophobic core, the lipid-water interfaces and the bulk water regions. In Fig. 2 we show the composition of the membrane as a function of the bilayer normal: methyl groups of the SDPC molecules define the location the hydrophobic core; carbonyl groups define the location of lipid-water interfaces; and phosphate groups define the location of the lipid heads. Beyond the lipid heads, the bulk water solvates the receptor extracellular domains. Hence, we consider that the receptor conformational dynamics was sampled in an equilibrated molecular environment that transitioned from the hydrophobic core up to the bulk water.

Fig 2. Mass distribution as function of the bilayer normal. Water and SDPC functional groups were identified in both the FSHR (top panel) and the LHCGR (bottom panel) systems. The calculations were performed for the last 40 ns of trajectory for replicates R1-R3. Distributions are normalized to the number of atoms/Å^3^.

To verify the receptors’ stability in the membrane environment, we calculated the root mean square displacements (RMSD) for C_α_ atoms of the transmembrane domains (Fig. 3). From the starting structure, RMSD reached a stable average below 3 Å in both the FSHR and the LHCGR; the corresponding fluctuations where consistent among all replicates. Hence, according with our strategy for detecting of conformational changes, the receptors’ dynamics was indeed modulated in a well-structured bilayer. Therefore, conformational fluctuations of the receptor responded to the membrane domains, from the hydrophobic core to solvent aqueous media.

Figure 3. Root mean squared displacement (RMSD) calculations for the C_α_ atoms of the transmembrane domains in the FSHR (top panel) and the LHCGR (bottom panel). Results for R1-R3 replicates were included.

Figures 1-3 were included as supplementary information in S2-S4 Figs.

As a potentially interesting point, the data obtained for the FSHR can be compared to the cryo-EM structure 8I2G obtained at 2.8 Å resolution to test if the AF structure refinement combined with MD can bring the system to a high-quality structure. Note that the HR domain in the AF structure used in the work is predicted at very low to low confidence level.

In Fig. 4 we show the structural alignment of our computational model for the FSHR and the cryo-EM structure PDB:8I2G in complex with the G_s_-protein and its cognate ligand (FSH) (not shown).

Fig 4. Structural alignment of the FSHR model (purple cartoons) and the cryo-EM structure of FSHR (orange cartoons) bound to FSH (not shown) and Gs-protein. As a context, in the background, lipid tails are depicted as white sticks; and phosphate atoms, as olive colored spheres. Cysteine atoms are also shown in licorice blue and cyan, respectively, for FSHR and 8I2G structures.

The overall length and orientation of the TM domains are similar in both structures (RMSD of 1.97 Å). The hinge α-helix (P272-N280), and the P10 loop (F350-Y359) at the interface between LRR and TM were well preserved, including the position of the disulfide bridges C276-C356 and C275-C346. Partial comparison of the HR region suggests that the model predicted larger α-helix segments, which were not possible to verify due to the missing residues (I296 to M328) in the cryo-EM structure. Table 1 shows distances among the FSHR domains LRR (L31), HR (S295) and TM (L460) found in the cryo-EM FSHR structure.

Table 1. Distances among C_α_ atoms of residues at the LRR, HR and HR domains.

| d (LRR-TM) | d(LRR-HR) | d(TM-HR) |
| --- | --- | --- |
| 89.37Å | 48.26Å | 76.17 Å |

In our trajectories, we found configurations of the FSHR sampling distances in intervals: 72< d(LRR-TM) < 93Å; 47< d(LRR-HR) <80A, and 80< d(TM-HR) <100 Å. Therefore, due to the flexibility of the extracellular domain, trajectories of the FSHR model populated configurations close to those of the resolved structure. Of course, the structural information from the cryo-EM FSHR is very useful to improve computational models and simulation protocols for a next stage of our research.

Fig 4 and table 1 were included, respectively, in Fig.1B and Table 1 of the main text.

As a final point, in both structures the LRR is glycosylated and the root of the N-glycans is resolved in both cryo-EM structures. These glycans should be reconstructed, both for completeness (both hormone receptors are glycoproteins) and because they may very well be functional, and thus useful to the general aims of the work.

As suggested, we set up a FSHR model glycosylated at N191; and LHCGR, at N195 and N299. Then, we generated additional ~150 ns trajectories. In Fig 5 we show the box dimensions for the N-glycan-asparagine receptors.

Fig 5. Box dimensions for the N-glycated receptors. Gray area corresponds to the interval in which 95% of the values were observed in the replicates without the glycan modifications.

Fig 6. Root mean squared deviation (RMSD) for the TM domains in the N-glycan-asparagine. A. FSHR glycated at N191. B. LHCGR glycated at N195 and N299. Gray areas represent the interval in which 95 % of RMSD values distribute in the replicates without glycan modifications.

According to previous reports [1-4], we introduced glycans only at confirmed positions in the human receptors. Fig 7 shows a projection of the PC1 on the structure of the receptors. The motion of the LRR and the HR was compared against that of replicate R2. Even though the trajectory for the glycated receptors was only of 150 ns, it seems that the glycation might have an effect on the overall dynamics of the receptor, an observation that deserves further studies to evaluate the structural fluctuations resulting from posttranslational modifications, which is beyond the purpose of the present study. Nevertheless, applying the proposed strategy to characterize the conformational space, would be very illustrative to find what are the conformations that can be stabilized as a result of glycation at particular residues. We thank the reviewer for bringing this issue up into discussion.

Fig 7. Projection of PC1 on the structure of N-glycosylated receptors. A. FSHR in white ribbons with a glycan side chain at position N191. B. LHCGR in with ribbons with two glycan side chains at positions N195 and N299. Arrows represent the direction and amplitude of the motion, in the glycated receptors and in the native structure replicate R2.

There are typos throughout that can be corrected with a careful reading of the draft.

Typos were corrected.

Reviewer #2: This study was performed to identify the conformational changes in FSHR and LHCGR using MD simulations. In these receptors, distances among the LRR, HR and TM domains were measured to determine a key region involved in the activation of the receptor. Finally, they have proposed that LRR-TM, or TM-HR relative distances may be useful to explore the transitional states between the active/ inactive states.

The proposed approach using MD simulation study to understand states between active and inactive seems a strategy with great potential. However, there are several substantial concerns that require the authors' attention.

1. The introduction section contains excessive information regarding PC analysis. In my opinion, it is unnecessary to provide a detailed description of PCs in this section; instead, it would be more appropriate to emphasize their significance within this study and include them as considered parameters in the methods section.

Description of PC analysis was omitted in the introduction. Description of PC analysis combined with cluster analysis was included in methods section, and their relevance for the present study in discussion section.

2. The authors have thoroughly discussed the displacement and rearrangement of helices 5-7 in GPCR. However, similar aspects regarding FSHR activation have not been discussed, despite their elucidation in a recent publication (<https://doi.org/10.1016/j.compbiomed.2023.106588>).

We thank reviewer for sharing a relevant reference for our study. We included calculations for the kink on helix 6 in the revised version, as it was a signature in conformational changes of the FSHR receptor with agonist molecule.

Fig 7. A. Kink angle of TM helix 6 in FSHR for replicates R1-R3. Calculation was performed using positions of C_α_ atoms of L597 and M858 for the upper half, and D581 and S565 for the lower half. B. Kink angle of TM helix 6 in LHCGR for replicates R1-R3. Calculation was performed using positions of C_α_ atoms of F594 and S586 for the upper half, and D578 and C563 for the lower half. The kink angle was calculated from the tilt of the upper half relative to the lower.

Fig 7 was included as S11 Fig in supplementary information.

3. In material and methods section, why were both Na+ and Cl- ions used in the second simulation box? It is not mentioned whether they were used to neutralize the system or to provide a salt concentration.

Three sodium ions were included in the FSHR simulation box to balance net charge of the receptor. In contrast, the net charge of LCGHR was balanced with four chloride ions with consideration of preserving the same amount of sodium ions as in the FSHR simulation box. Salt concentrations were 3.5 and 8 mM, respectively, for FSHR and LHCGR systems.

Ion concentration of the simulation boxes was included in methods section.

4. The statement is written as “The chain 612 R (segment PROD) of the pdb file was selected to be inserted in a POPC lipid bilayer”. Why was this performed, its not clear in the statement.
5. In line 612 statement, "The chain 612 R (segment PROD) of the pdb file was selected to be inserted in a POPC lipid bilayer," lacks clarity regarding the reason for this action.

We use the chain R because it corresponds to the receptor. The PDB contains coordinates for the FSH hormone (chains X Y), and the G-protein trimer (chains A,B,G). In the revised version we now clarify the origin of the R chain selection.

6. In the materials and methods section, two different types of systems are described only for LHCGR, but the importance of these two considered systems is not adequately explained.

We consider that the membrane lipids could play a role in the flexibility of the TM domains, as it was reported for the rhodopsin light receptor [5, 6]. We initially set up the LHCGR because it was cryo-EM resolved structure, thus simulation results could be directly compared with a known structure while the FSHR was an AF2 model. Now the structure of the FSHR is also available and both systems can be compared in similar conditions. For the purpose of the present MD protocol, we use the use the POPC set up of LHCGR to harvest configurations that were eventually relaxed in the SDPC bilayer, as procedure to enhance the exploration of the conformational landscape. In the revised manuscript we included sentence regarding the purpose of relaxing the TM domain in polyunsaturated lipids (in line 657-659):

By changing the lipid environment, from POPC to SDPC, we consider the possibility of enhancing the dynamics of the TM domain in poly-unsaturated lipid tails, as reported on the influence of DHA lipids in the rhodopsin functions [5,6]

7. It is not mentioned whether FSHR/LHCGR was simulated with or without a cognate ligand. Without this information, it is unclear how the transition between active and inactive states was expected if FSHR/LHCGR was simulated without a cognate ligand.

None of the receptors was simulated with its cognate ligand. At this stage of our research we did not pursue complete active to inactive transitions, as commented by the reviewer. In fact, we explored the receptors’ fluctuations involved by random thermal motion and molecular interactions stabilizing different conformations compatible with the active state. On the other hand, it is well known that for GPCRs fluctuations between the active and inactive states may occur in the absence of ligand, as it occurs in constitutive activation, and what the ligand does is to stabilize a given (ie. inactive, intermediate or active) conformation [7-9]. The data presented in our paper might be useful to characterize the effects of small molecule allosteric modulators in stabilizing particular conformation(s) of mutant FSHRs to correct their intracellular traffic to the plasma membrane and provoke biased signaling in the absence of ligand, an ongoing project applying different experimental approaches, including in silico strategies.

8. In this manuscript, the results are exclusively presented in the form of changes in distances among LRR, HR, and TM. It is also crucial to depict the structural changes occurring in these regions for both receptors. It would be interesting if the authors could visually represent the movements and shifts in these domains and then compare the domain movements between FSHR and LHCGR.

A new figure showing a projection of the first principal component (PC1) over FSHR and LHCGR structures was generated. In Fig 8, the motion of the C_α_ atoms at the LRR, HR and TM domains is represented as arrows. The size of the arrows corresponds to the amplitude of the motion. Because of the rigid core of the TM domain, the LRR and HR display large amplitudes of motions and in different directions in the R1-R3 replicates.

Fig 8. Projection of the first principal component (PC1) on the FSHR and the LHCGR, both depicted in orange. Arrows sizes correspond to the amplitude of the motion detected in PC1, with color code: R1-yellow, R2-white, R3-purple. In FSHR and LHCGR, the HR motion was very dynamic in replicates R1-R3; in LHCGR the LRR motion showed different direction and amplitudes in R1-R3, while in FSHR the LRR motion had similar tendency of motion among the R1-R3 replicates. The TM domain was the rigid core on both receptors.

Motion of the LRR and HR in LHCGR was rather orthogonal among the replicates, which suggest that R1-R3 trajectories sampled distinct configurational regions in contrast to the FSHR. The relative wider sampling in LHCGR can be explained by the consideration of using initial configurations harvested from the POCP run at 0, 100 and 180 ns; therefore, implementation MD studies for exploring the potential energy landscape may profit from generation of multiple independent trajectories and also starting with different configurations. The weighted ensemble simulation could be very useful for the proposes of searching systematically conformational changes from multiple trajectories and initial states.

9. Authors have discussed that effective motion of the ECD was observed which is important for the activation of receptors. From the manuscript, it appears that this study did not consider agonist-complexed FSHR/LHCGR. If that is the case, how can we expect movement in the ECD to correspond to receptor activation?

Motion of the ECD was detected in the active conformation of the FSHR, and LHCGR, which was the starting conformation. The RMSD of the TM domains in the model FSHR and the resolved structure, bound to Gs-protein and its ligand, was 1.97 Å; the LHCGR was also taken form the active state of the receptor PDB:7FII. Therefore, conformations generated by MD trajectories were compatible with the active state in both receptors. The ECD still displayed a great deal of mobility as a result of interactions with the membrane, aqueous media. and thermal fluctuations.

A few minor suggestions:

1. The title should be concise.

Title was changed to “Tracking conformational transitions of the gonadotropin hormone receptors in a bilayer of (SDPC) poly-unsaturated lipids from all-atom molecular dynamics simulations”.

2. Replace THS with TSHR in the abstract.

Done

3. The abbreviations used in Table 1 appear to be incorrect.

Corrected

4. In line 573, please verify the acronym "EC2" and replace it with the correct one.

We thank the reviewer for pointing this inconsistency. We in fact included a brief discussion on the role of the hinge α-helix in signal transduction upon ligand binding (lines 579-587).

5. The manuscript needs to be carefully reviewed for grammatical errors.

Corrected.

**Have the authors made all data and (if applicable) computational code underlying the findings in their manuscript fully available?**
The [PLOS Data policy](https://journals.plos.org/ploscompbiol/s/materials-and-software-sharing) requires authors to make all data and code underlying the findings described in their manuscript fully available without restriction, with rare exception (please refer to the Data Availability Statement in the manuscript PDF file). The data and code should be provided as part of the manuscript or its supporting information, or deposited to a public repository. For example, in addition to summary statistics, the data points behind means, medians and variance measures should be available. If there are restrictions on publicly sharing data or code —e.g. participant privacy or use of data from a third party—those must be specified.

Reviewer #1: **No:** I haven't seen any electronic data in addition to the manuscript.

Reviewer #2: Yes

PLOS authors have the option to publish the peer review history of their article ([what does this mean?](https://journals.plos.org/ploscompbiol/s/editorial-and-peer-review-process#loc-peer-review-history)). If published, this will include your full peer review and any attached files.


If you choose “no”, your identity will remain anonymous but your review may still be made public.


**Do you want your identity to be public for this peer review?** For information about this choice, including consent withdrawal, please see our [Privacy Policy](https://www.plos.org/privacy-policy).

Reviewer #1: No

Reviewer #2: **Yes:** Chandan Kumar

Figure Files:

While revising your submission, please upload your figure files to the Preflight Analysis and Conversion Engine (PACE) digital diagnostic tool, [https://pacev2.apexcovantage.com](https://pacev2.apexcovantage.com/). PACE helps ensure that figures meet PLOS requirements. To use PACE, you must first register as a user. Then, login and navigate to the UPLOAD tab, where you will find detailed instructions on how to use the tool. If you encounter any issues or have any questions when using PACE, please email us at [figures@plos.org](mailto:figures@plos.org).

Data Requirements:

Please note that, as a condition of publication, PLOS' data policy requires that you make available all data used to draw the conclusions outlined in your manuscript. Data must be deposited in an appropriate repository, included within the body of the manuscript, or uploaded as supporting information. This includes all numerical values that were used to generate graphs, histograms etc.. For an example in PLOS Biology see here: <http://www.plosbiology.org/article/info%3Adoi%2F10.1371%2Fjournal.pbio.1001908#s5>.

Reproducibility:

To enhance the reproducibility of your results, we recommend that you deposit your laboratory protocols in [protocols.io](http://protocols.io), where a protocol can be assigned its own identifier (DOI) such that it can be cited independently in the future. Additionally, PLOS ONE offers an option to publish peer-reviewed clinical study protocols. Read more information on sharing protocols at <https://plos.org/protocols?utm_medium=editorial-email&utm_source=authorletters&utm_campaign=protocols>

**References**

1.	Fan QR, Hendrickson WA. Structure of human follicle-stimulating hormone in complex with its receptor. Nature. 2005;433:269–77

2.	Jiang X, Liu H, Chen X, Chen P-H, Fischer D, Sriraman V, et al. Structure of follicle-stimulating hormone in complex with the entire ectodomain of its receptor. Proc Nat Acad Sci USA. 2012;109:12491–6. doi: <https://doi.org/10.1073/pnas.1206643109>.

3.	Ascoli M, Fanelli F, Segaloff DL. The lutropin/choriogonadotropin receptor, a 2002 perspective. Endocrine reviews. 2002;23(2):141-74. doi: <https://doi.org/10.1210/edrv.23.2.0462>.

4.	Ulloa-Aguirre A, Zariñán T. The follitropin receptor: matching structure and function. Molecular pharmacology. 2016;90(5):596-608. doi: <http://dx.doi.org/10.1124/mol.116.104398>.

5.	Teague Jr WE, Soubias O, Petrache H, Fuller N, Hines KG, Rand RP, et al. Elastic properties of polyunsaturated phosphatidylethanolamines influence rhodopsin function. Faraday Discuss. 2013;161:383-95.

6.	Brown MF. Modulation of rhodopsin function by properties of the membrane bilayer. Chemistry and Physics of Lipids. 1994;73:159-80.

7.	Hilger D, Masureel M, Kobilka BK. Structure and dynamics of GPCR signaling complexes. Nature structural & molecular biology. 2018;25(1):4-12. doi: <https://doi.org/10.1038/s41594-017-0011-7>.

8.	De Lean A, Stadel J, Lefkowitz RJ. A ternary complex model explains the agonist-specific binding properties of the adenylate cyclase-coupled beta-adrenergic receptor. Journal of Biological Chemistry. 1980;255(15):7108-17.

9.	Ulloa-Aguirre A, Reiter E, Bousfield G, Dias JA, Huhtaniemi I. Constitutive activity in gonadotropin receptors. Adv Pharmacol. 2014;70:37-80. Epub 2014/06/17. doi: 10.1016/B978-0-12-417197-8.00002-X. PubMed PMID: 24931192.
