# Supplementary material for: Tracking conformational transitions of the gonadotropin hormone receptors in a bilayer of (SDPC) poly-unsaturated lipids from all-atom molecular dynamics simulations
Source: PLoS Comput Biol. 2024 Jan 11;20(1):e1011415. doi: 10.1371/journal.pcbi.1011415 (PMC10807830; doi:10.1371/journal.pcbi.1011415)
Supplement: Attachment — Submitted filename: Answers to reviewer.docx [file pcbi.1011415.s015.docx]

**Answers to reviewer**

Reviewer #2: The revised manuscript shows improvement with the implemented modifications, and almost all concerns have been addressed. While the manuscript is now more comprehensible, certain issues still require resolution.

Questions:

1. Authors have calculated the kink at helix 6 (H6) in both FSHR and LHCGR and discussed the importance of H6 fluctuations for receptor activation. It appears from Figure S11 that the angle at H6 of FSHR increased, while in LHCGR, the angle decreased over the simulation time period. However, the observed differences for these two receptors are not discussed in the manuscript.

**Answer:**

Thanks for this interesting comment. In the revised version, the kink angle calculation of TM6 was performed relative to the angle of the crystal structures. This measure was more appropriate to determine the adjustment of the structure in the membrane environment instead of comparing absolute values. Thus, the kink of TM6 in the FSHR remained close to the value of the crystal structure with averages of -2.02±1.12, 5.64±0.77 and 4.03±1.49 for the last 164 ns in runs R1, R2, and R3, respectively; in the LHCGR, averages were slightly higher in R2 and R3 (also in the last 164 ns), of 7.82±1.48 and 8.71±2.02, respectively. Still the kink angles were comparable in both receptors since the reference value was 44 degrees in the FSHR, higher than the reference value of 37 degrees in the LHCGR. With the calculation of the relative kink angle, the apparent decreasing angle remained within fluctuations of the relative kink angle of TM6 in both receptors. Description of this observation are now included in the revised version of the MS (lines 589-596); S11 Fig in SI information was updated, and S12 Table for the kink angle averages was added.

S11 Fig. Kink angle value for TM6 in FSHR (A) and LHCGR (B).

2. In my previous comment, number 3, the statement regarding neutralizing and salt concentration for the simulation systems is now justified through the response. However, the statement in the manuscript is not very clear.

**Answer:**

The sentence on the addition of neutralizing ions has been modified in the new version of the ms (lines 635-637).

3. Now it is very clear that both the receptors were simulated without their cognate ligands. However, some statements in the text may be confusing (eg. Line 55-59 in revised manuscript), and it needs clarification that the receptors were not simulated with cognate ligands. Instead, the study focused on exploring various receptor conformations through random thermal motion.

**Answer:**

The sentence was modified in the revised manuscript (lines 55-58, new version).
